# A rare presentation of Pott's disease with bilateral psoas abscess during pregnancy in a resource-limited setting: Case report from a resource-limited setting

**DOI:** 10.1016/j.ijscr.2025.111312

**Published:** 2025-04-17

**Authors:** Wali Ahmed Nur, Dagne Aschenaki Argaw, Musse Ahmed Ibrahim, Abdirahman Ahmed Abdulahi, Mohamed Ayanle Hassan, Addisu Assfaw Ayen

**Affiliations:** a Gerbo primary hospital, Somalia, Ethiopia; b Department of General Surgery, Gerbo Primary Hospital, Somalia, Ethiopia; c Department of General Surgery, Jigjiga University, Jigjiga, Ethiopia; d Somalia regional health bureau, Jigjiga, Ethiopia; e Department of Internal Medicine, Debre Tabor University, Debre Tabor, Ethiopia

**Keywords:** Psoas TB abscess, Extra pulmonary TB, Pott's disease, Case report, Ethiopia

## Abstract

**Introduction and importance:**

TB remains a global health threat, especially in low-income countries. Spinal TB diagnosis in pregnancy is challenging, leading to potentially severe complications like psoas TB abscess.

**Case presentation:**

A 30-year-old multiparous woman from Somalia region, Ethiopia in her second trimester of pregnancy who was diagnosed with isolated Pott's disease complicated by bilateral large Psoas abscesses. The diagnosis was made using lumbosacral X-ray, abdomino-pelvic ultrasound, and ultrasound-guided aspiration confirming tuberculosis. After she presented with a four-year history of chronic lower back pain, exacerbated by lower back and left lower quadrant swelling for six months, accompanied by constitutional symptoms. The patient was successfully treated with anti-tubercular therapy with no complication to her and her baby.

**Discussion:**

Pott's disease with tuberculous psoas abscess is rare and challenging in pregnancy, often causing complications. Hormonal changes mask symptoms, delaying diagnosis. While *Staphylococcus aureus* is common, *Mycobacterium tuberculosis* is relevant in developing countries with limited diagnostics. Pott's disease constitutes 50 % of skeletal TB, with psoas abscesses in 5 % and rare bilateral presentations. Treatment involves safe, prolonged anti-TB therapy (2RH/10RHZE with pyridoxine), and delivery mode depends on obstetric factors.

**Conclusion:**

Pott's disease with TB psoas abscess, while rare, can occur in pregnancy despite immunocompetence. Early diagnosis in resource-limited settings and appropriate treatment are essential for good maternal and child health.

## Highlights

• Pott's disease presenting with bilateral psoas abscesses;a rare presentation of Extra-pulmonary TB in immunocompetent individuals
• Bilateral psoas abscesses in pregnancy is a very rare complication pott’s disease.
• Challenges in resource-constrained environments in terms of diagnosis and management.
• Initiating appropriate anti-TB treatment early in the course of the disease is crucial for improving patient outcomes both for mother and baby.

## 1 Introduction

The 2024 Global Tuberculosis Report identifies tuberculosis (TB) as a major global health challenge, disproportionately impacting low- and middle-income countries (1). Worldwide, the report estimates a TB incidence of 134 per 100,000 population, with a total of 10.8 million new cases (1). While TB typically presents with pulmonary symptoms, approximately 10 % of cases involve extrapulmonary manifestations (2). Of these extrapulmonary TB cases, about 2 % affect the spine (2). Diagnosing spinal TB during pregnancy can be challenging due to the common occurrence of non-specific lower back pain associated with physiological changes (3). Tuberculous psoas abscess complicating pregnancy is a rare condition that is infrequently reported in the literature (4). Delayed diagnosis of Pott's disease can lead to rare complications, such as psoas TB abscesses, which pose a significant burden to both mother and fetus and may result in permanent disabilities, including spinal cord compression (3,5). Diagnosing Pott's disease requires spinal MRI, which is considered safe during pregnancy (6). However, assessment of associated psoas TB abscesses may necessitate abdominopelvic CT, a modality generally avoided in pregnancy (6). Consequently, diagnosing Pott's disease complicated by psoas abscess in pregnancy can be challenging, particularly when both imaging modalities are not readily available as in our setup. We report a case of isolated Pott's disease with bilateral abscesses in a pregnant woman, diagnosed using limited resources and successfully treated with anti-TB therapy. This case report adheres to the Surgical Case Report (SCARE) 2023 guidelines (7).

## 2 Case presentation

A 30-year-old G8P7 woman from Somalia, Ethiopia, approximately 6 months amenorrheic without prenatal care, presented with a one-month history of worsening left lower quadrant pain and swelling on April 2024. The patient reports a similar, less severe, unilateral swelling and pain in the same location for the past 6 months, which has progressively worsened and become bilateral in the past month. Associated symptoms include fever, loss of appetite, and night sweats, though the patient denies significant weight loss, possibly due to her pregnancy. She also reports a four-year history of chronic lower back pain and a six-month history of lower back swelling (lumbar area). The patient denies any history of trauma, prior radiation exposure, known chronic medical illnesses, cough, shortness of breath, contact with aborts material, similar illnesses in her vicinity, or prior radiation exposure.

**Fig. 1:**
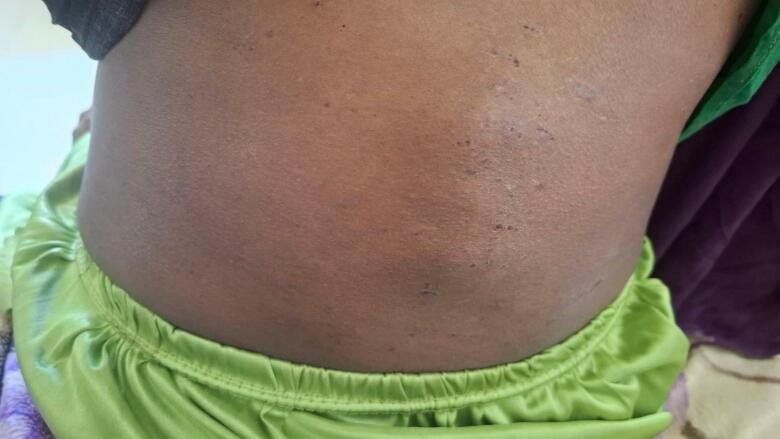
Showing gibbus deformity.

On physical examination, the patient was conscious and appeared acutely ill (in pain), with a chronically unwell appearance. She was not in cardiorespiratory distress. Vital signs revealed a pulse rate of 110 bpm and a temperature of 38.5 °C; other vital signs were within normal limits. Chest auscultation was clear. Abdominal examination revealed a 26-week size, gravid uterus and a 10 × 4 cm tender mass in the left lower quadrant. Palpation of the lumbar area revealed a hard, non-tender mass with a gibbus deformity (as shown in Fig. 1). No other pertinent findings were noted on physical examination.

Laboratory investigations revealed severe anemia (hemoglobin 7 g/dL, MCV 65.8 fL), elevated inflammatory markers (ESR 110 mm/h, WBC 10,690/μL), and negative screening tests for hepatitis B, retrovirus, and syphilis. Serum electrolytes, chest x-ray, and organ function test were within normal limits.

**Fig. 2:**
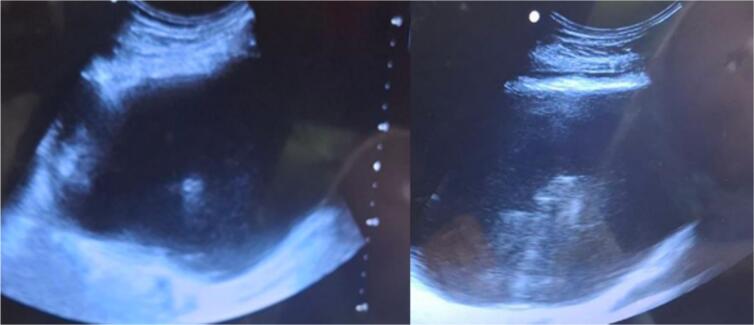
Abdominal ultrasound demonstrated evidence of bilateral psoas abscesses.

Abdominal and pelvic ultrasound revealed complex fluid collections measuring 12x8x6 cm in the right psoas muscle and 17x12x8 cm in the left psoas muscle, consistent with bilateral psoas abscesses (as shown in Fig. 2).

**Fig. 3:**
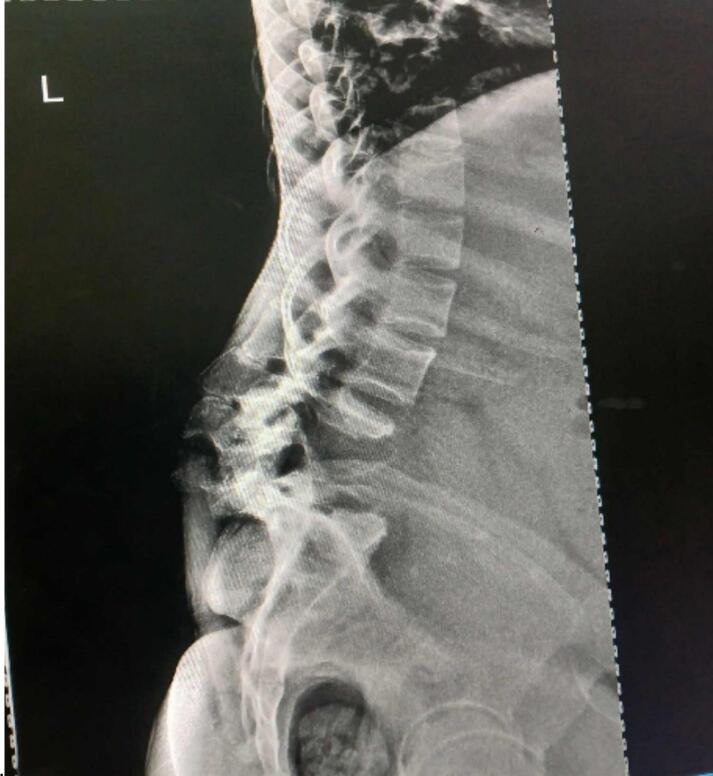
Lumbar spine X-ray demonstrated severe kyphotic deformity and destruction of the lower lumbar vertebrae (L4-L5), with wedging and sclerosis of L3 and a perivertebral soft tissue mass.

Lumbar spine X-ray revealed severe kyphotic deformity of the lower lumbar spine with complete destruction and collapse of the L4 and L5 vertebral bodies. Anterior wedging and sclerosis of the L3 vertebral body, along with an adjacent perivertebral soft tissue mass, suggest TB spondylitis with prevertebral abscess (as shown in Fig. 3). Lumbosacral MRI was not performed due to lack of availability.

Ultrasound-guided aspiration of the psoas muscle yielded 50 cc of cloudy, pale yellow fluid. GeneXpert testing was positive for *Mycobacterium tuberculosis* (TB) with no rifampicin resistance. Further analysis showed a negative AFB stain, negative gram stain, and a cell count of 1000 cells, 70 % of which were lymphocytes.

Then we diagnosed her with isolated Pott's disease, complicated by bilateral large TB abscesses and a second-trimester pregnancy, the patient was initiated on antitubercular therapy (ATT) per WHO and national guidelines (2 RHZE/10RH) with pyridoxine supplementation at the end of April 2024. Ante-natal care (ANC) was continued throughout. At two weeks, subjective improvements (reduced fever, improved appetite, and general wellbeing) were noted despite persistent psoas collections. Follow-up at one month showed a decrease in psoas collections, which resolved completely after four months of treatment. She delivered without complications in early June 2024 and is currently continuing anti TB with good overall progress and will complete her anti TB on end of April 2025.

## 3 Discussion

Pott's disease with a TB psoas abscess is a rare and challenging condition in pregnancy, often leading to obstetric and general complications (4). Delayed diagnosis, due to hormonal and mechanical changes that can mask symptoms like lower back pain, contributes to this complexity (8). Pott's disease, a form of skeletal tuberculosis, accounts for 50 % of skeletal TB cases (9). Of these, psoas TB abscesses occur in approximately 5 %, with bilateral presentations being rare (9).

Although *Staphylococcus aureus* is the most common cause of psoas abscesses, *Mycobacterium tuberculosis* remains a relevant pathogen, particularly in developing countries. The diagnosis of tuberculous psoas abscesses in these regions is challenging due to limited diagnostic capabilities and delayed patient presentation (10). Secondary psoas abscesses are the predominant type. This patient's psoas abscess was a complication of Pott's disease. Although Crohn's disease is often mentioned in the literature as a cause of psoas abscess, spinal pathology, particularly Pott's disease, represents a significant source of secondary psoas abscesses (11). While secondary tuberculous psoas abscesses from spinal TB are common and challenging in developing countries, they are typically unilateral and small. The large, bilateral secondary tuberculous psoas abscess seen in our patient is exceedingly rare, representing approximately 3 % of secondary psoas abscesses (12).

Spinal TB with psoas abscess is more prevalent in patients with acquired or congenital immunodeficiency, those receiving immunosuppressive agents (e.g., chemotherapy, steroids, or biologics), and those with chronic medical illnesses (13). Although our patient had no identified risk factors for psoas abscess, pregnancy-related immunosuppression may have contributed to this rare presentation. The literature on tuberculous psoas abscess in pregnancy is sparse, reflecting its rarity (4). One such report, by Dagar Mamta et al. (2009), details a 21-year-old woman diagnosed with spinal TB and psoas abscess at 15 weeks of gestation. The patient was successfully treated with antitubercular medications (14). In contrast, a 30-year-old immunocompetent male patient at the same hospital was diagnosed with Pott's disease and bilateral psoas abscesses (15). However, the abscesses in this patient were not as large as those observed in the pregnant patient.

Our patient presented with prolonged lower back pain, fever, and swelling associated with left lower quadrant pain. These symptoms are commonly reported in Pott's disease, with or without complications such as spinal cord compression or deformity and psoas abscess is another recognized complication (16). A literature review of psoas abscess in pregnancy, involving ten cases, found that the typical gestational age at presentation ranged from 13 to 39 weeks (17). Our patient presented within this range, at 26 weeks of gestation (second trimester).

Due to the patient's delayed presentation and the late diagnosis, Pott's disease with psoas abscess carries risks for both mother (e.g., malnutrition, neurologic complications, sepsis) and fetus (e.g., preterm delivery, neonatal sepsis, growth restriction) (5). Fortunately, our patient experienced no such complications.

Diagnosing Pott's disease with psoas abscess during pregnancy necessitates basic investigations and bacteriological confirmation (18). Our patient tested positive for *Mycobacterium tuberculosis* via GeneXpert analysis of the psoas collection, and lumbosacral X-ray revealed vertebral pathology. Although CT scans are generally contraindicated during pregnancy, MRI is a safe alternative for diagnostic imaging and assessing disease extent (19). However, neither MRI nor CT scan was available for our patient. In resource-rich settings, diagnostic imaging for Pott's disease and psoas abscess during pregnancy often prioritizes methods that minimize radiation exposure like MRI (6). However, in resource-limited settings, where advanced imaging techniques may not be readily available, X-ray may be considered a necessary alternative, particularly in the later trimesters, when the risk to the fetus is reduced; the risk of X-ray exposure, should be weighed against its diagnostic value and accuracy (6).

Once Pott's disease with psoas abscess is confirmed during pregnancy, treatment mirrors that of the general population, typically involving prolonged anti-tuberculosis therapy (2RH/10RHZE with pyridoxine) (20). All recommended drugs are considered safe for use during pregnancy. Although our patient did not exhibit surgical indications such as spinal cord compression, surgical intervention may be necessary in some cases (21). The mode of delivery in patients with Pott's disease and psoas abscess is determined by obstetric indications, as both cesarean and vaginal delivery have similar outcomes (17).

Although rare, tuberculous psoas abscess should be considered in resource-limited settings. Patient outcomes can be positively impacted through modifiable factors such as increased community awareness promoting early presentation, enhanced healthcare professional training in basic imaging interpretation, and improved diagnostic infrastructure.

## 4 Conclusion

Tuberculosis remains a significant infectious health challenge, particularly in developing countries, with diverse pulmonary and extrapulmonary manifestations. Although immunocompromise increases the risk of Pott's disease with psoas TB abscess—a rare presentation of TB—it can also occur in immunocompetent individuals, though rarely in pregnancy. In resource-constrained settings, delayed presentation can lead to psoas abscess complications, necessitating a high index of suspicion for diagnosis. Timely and appropriate treatment of Pott's disease with psoas abscess in pregnancy can result in favorable outcomes for both mother and child.

## Abbreviations

AFB: Acid-fast bacillus
TB: Tuberculosis
2RHZE: Isoniazid, Rifampin, Ethambutol, and Pyrazinamide for 2 months
10RH: Rifampicin and Isoniazid for 4 months
WBC: white blood cell
WHO: World Health organization

## Consent

Written informed consent was obtained from the patient for publication and any accompanying images. A copy of the written consent is available for review by the Editor-in-Chief of this journal on request.

## Ethical approval

Ethical approval for this study was provided by our institution ethical review committee.

## Guarantor

Addisu Assfaw Ayen.

## Research registration number

N/A

## Declaration of Generative AI and AI-assisted technologies in the writing process

AI language modelling tools were utilized for the improvement of English-language only in this case report.

## Funding

There is no source of funding found for this paper.

## Author contribution

AAA^1^: Conceptualization, design of the study, acquisition of data, drafting the article, revising it critically for important intellectual content, approval of the version to be submitted.

WAN: Analysis, interpretation of data, drafting the article, revising it critically for important intellectual content, approval of the version to be submitted.

DAA: Conceptualization, analysis, drafting the article, revising it critically for important intellectual content, approval of the version to be submitted.

MAI: Acquisition of data, analysis, revising it critically for important intellectual content, approval of the version to be submitted.

AAA^4^: Acquisition of data, analysis, revising it critically for important intellectual content, approval of the version to be submitted.

MAH: Acquisition of data, analysis, revising it critically for important intellectual content, approval of the version to be submitted.

## Declaration of competing interest

All authors declare that they have no conflict of interest.
